# Fabrication of Triethylenetetramine Terminal Hyperbranched Dendrimer-Like Polymer Modified Silica Gel and Its Prominent Recovery Toward Au (III)

**DOI:** 10.3389/fchem.2019.00577

**Published:** 2019-08-14

**Authors:** Ying Zhang, Rongjun Qu, Ting Xu, Yu Zhang, Changmei Sun, Chunnuan Ji, Ying Wang

**Affiliations:** School of Chemistry and Materials Science, Ludong University, Yantai, China

**Keywords:** silica-gel, triethylenetetramine, dendrimer-like highly branched polymer, Au(III), adsorption

## Abstract

To further increase the quantity and density of functional groups on adsorbent, terminal triethylenetetramine hyperbranched dendrimer-like polymer modified silica-gel (SG-TETA and SG-TETA2) was synthesized. The hyperbranched dendrimer-like polymer was successfully introduced onto silica gel and new cavities were formed, which was demonstrated by FTIR, SEM, and BET. The highest adsorption capacities of SG-TETA and SG-TETA2 obtained from Langmuir model toward Au(III) were 2.11, and 2.27 mmol g^−1^, respectively, indicating that SG-TETA2 possessing more functional groups had a better adsorption ability. Moreover, the adsorbents combined with Au(III) ion through chelation and electrostatic attraction mechanism, after which reduction reactions for Au(III) ion loaded on adsorbents proceeded. SG-TETA2 had better adsorption selectivity than SG-TETA in removing Au(III) in Au-existed ion solution systems. SG-TETA2 had higher overall adsorption capacities compared to silica-gel-based hyperbranched polymers functionalized by diethylenetriamine. Therefore, the effective recovery makes SG-TETA2 a practical adsorbent in removing Au(III) ion from e-wastes and industrial effluents with much prospect.

## Introduction

Gold (Au) is an indispensable and important precious metals that performs many functions, as it is one of the raw materials commonly found in electronics, catalysts, anti-corrosion materials (Das, 2010; Chen et al., 2015; Vojoudi et al., 2017). The extraction and recovery of Au(III) are not easy because the golden content in environmental, geological, and electronic materials is very low. With the industrial demand and its value rising steeply in recent years, the economic incentive to increase recycling or recovery of Au(III) from waste solutions has risen as well (Zhang et al., 2017). A variety of techniques such as chemical precipitation (Soylak and Tuzen, 2008; Boudesocque et al., 2014), membrane separation (Kargari et al., 2006; Wang et al., 2017), solvent extraction (Vidhate et al., 2015), and adsorption (Zhen et al., 2016; Tofan et al., 2017; Yu and Fein, 2017) have been introduced into enrich, and recover gold from waste water. Among these methods, the adsorption method has been evaluated as the most effective way, with attractive features such as easy operation, low-cost, and is particularly suitable for recovering the low level of gold which exists in industry wastewater.

Stable performance of matrix and effective chelating groups are the basic conditions of promising adsorbents. Nitrogen-containing adsorbents are commonly used as one of the most effective adsorbents for recovering Au(III). Plenty of designed adsorbents with high nitrogen content have been applied to selectively adsorb Au(III) from aqueous solution (Ahamed et al., 2013; Ebrahimzadeh et al., 2013; Tofan et al., 2017). Theory and empirical study indicate that nitrogen-containing adsorbents possess not only high adsorption capacities but excellent selectivity for binding Au(III).

The poly(amidoamine) (PAMAM) dendrimers exhibit excellent adsorption performance, relying on its unique three-dimensional steric configuration, and nanoscale shape, and topography exactly matching high density nitrogen-containing sites. The hyper-branched structures, lots of cavities and the presence of multiple functional groups between the branches can be useful in trapping other molecules (Vunain et al., 2016; Sajid et al., 2018). PAMAM dendrimers contain a high density of nitrogen ligands belonging to nitrogen functional groups such as amines and amides, and have strong chelating affinity with metal ions (Zarghami et al., 2016; Hayati et al., 2017; Ma et al., 2017). The PAMAM dendrimers are typically fabricated via repeated two-step process including Michael addition reaction and amidation with methyl acrylate (MA) and ethylene diamine (EDA). Notably, ethylene diamine is the most common choice for the repeating reaction to increase the nitrogen-containing functional groups. However, there are some other polyethylene polyamines, including diethylenetriamine (DETA), triethylenetetramine (TETA), tetraethylenepentamine (TEPA), besides EDA which might be a better option, which have more nitrogen active sites. Our group (Zhang et al., 2015) has been trying to prepare a PAMAM dendrimer-like polymer adsorbent using DETA as a reaction unit by combination of homogeneous and heterogeneous methods, and found the synthetic adsorbents had great adsorption for Au(III) influences. It's also important to note that it was difficult to obtain prospective nitrogen content on dendrimers through a completely “heterogeneous method” because steric hindrance generated from the grafted dendrimers (Qu et al., 2006). Although the completely “homogeneous method” was improved a lot, the purification progress is hard to operate (Qu et al., 2014). Meanwhile, no one had published about using long-chain TETA as a reaction unit for the synthesis of highly branched PAMAM polymer adsorption materials.

In this work, triethylenetetramine terminal hyperbranched dendrimer-like polymer modified silica gel adsorbents were synthesized based the prepared method of PAMAM, still with the combination of homogeneous method, and heterogeneous method. The objectives of this work were to probe the influences of long-chain TETA on the adsorption properties of the sort of adsorbents. More importantly, the selective adsorption performance, and adsorption mechanic for Au(III) were given.

## Materials and Methods

### Materials

Spherical silica-gel (particle size: 60–100 mesh) was obtained from Aladdin and activated as reported (Zhang et al., 2009). γ-Chloropropyltrimethoxysilane (CPTS) was bought from Qufu wanda chemical co. and used as received. MA and TETA were analytical reagent grade. HAuCl_4_·4H_2_O was purchased from Sinopharm Chemical Reagent Co.

### Instruments and Apparatus

The morphology was taken using a SU8010 (Hitachi, Japan). FTIR spectra were reported using a FTIR spectrophotometer Nicolet iS50 (Nicolet, American). Elemental analysis was obtained from the Elementar VarioEL III instrument, Elementar Co., Germany. The nitrogen adsorption–desorption isotherms were performed using ASAP Micromeritics (2020, USA). X-ray photoelectron spectroscopy were performed on ESCALAB Xi^+^ (Thermo Fisher Scientific, American). The concentrations of metal ions were supplied on the atomic absorption spectrometer (VARIAN AA240, American).

### Preparation of SG-TETA, and SG-TETA2

**Scheme 1:**
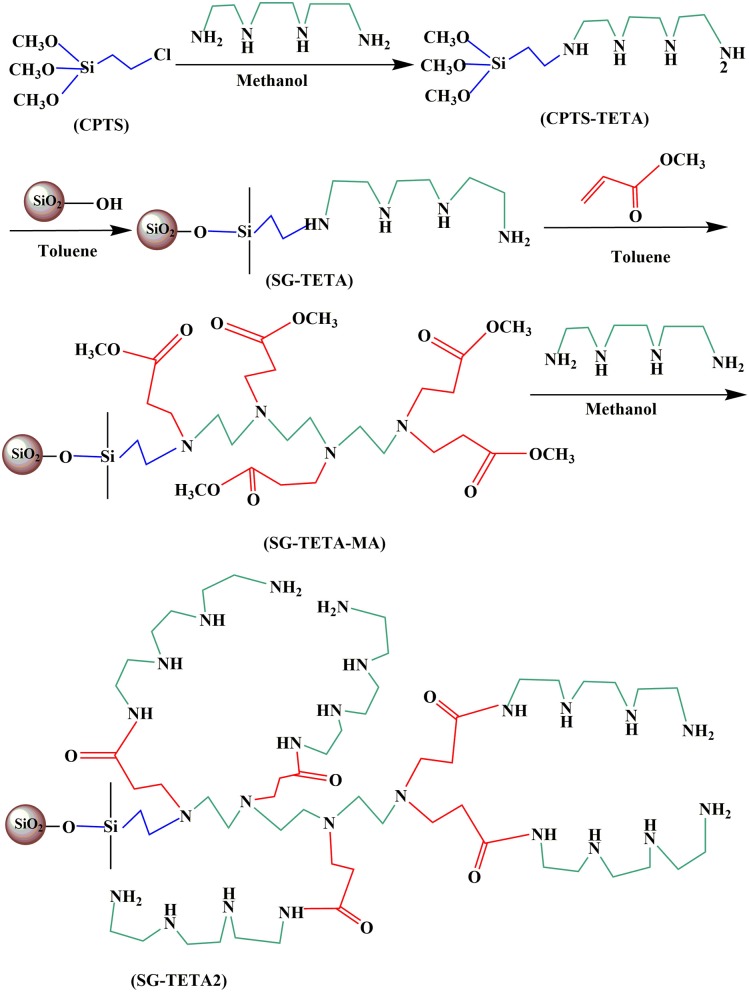
The synthesis schematic of SG-TETA and SG-TETA2.

The synthetic process of silica-gel-based dendrimer-like polymers are illustrated in Scheme 1. CPTS-TETA were obtained by according to our previous work (Zhang et al., 2009). CPTS (15 mL, 82.0 mmol) was refluxed with TETA (25 mL, 167.0 mmol) in methanol of 150 mL under protection of nitrogen about 12 h. Then CPTS-TETA was obtained through evaporation of solvent methanol. Next, CPTS-TETA was mixed with 15.0 g of activated silica gel in 150 mL of toluene under mechanical stirring and a nitrogen atmosphere. After continuous shaking under a reflux condition for 12 h, the target product first generation dendrimer adsorbent SG-TETA was extracted in turn with toluene and ethanol, and dried at 50°C for 72 h. The SG-TETA (6.6 g) and MA (16 ml, 176 mmol) were further dispersed in 50 mL methanol for 72 h at 50°C. Then the SG-TETA-MA was produced via filtering and extracting by methanol over 24 h. Next, the SG-TETA-MA (3.0 g) was treated with 60 ml (402 mmol) of TETA in 100 mL of methanol at 50°C for 5 days in water bath. Finally, the second generation dendrimer adsorbent SG-TETA2 was obtained after filtrating, washing and drying.

### Adsorption Study

The adsorption data was evaluated by the atomic absorption spectrometer. Each adsorption was repeated 3 times and the adsorption data averaged. The adsorbed Au(III) at equilibrium were obtained by calculating as follows:

(1) $$\begin{array}{r} q_{e}=\frac{\left(C_{0}-C_{e}\right)V}{m} \end{array}$$

Here *q*_*e*_ (mmol g^−1^), *C*_0_ (mmol L^−1^), and *C*_*e*_ (mmol L^−1^) are adsorption amount, initial and equilibrium, *m* (g) is the amount of adsorbent, and *V* (L) is the volume of the Au (III) solution.

After adsorption, the adsorbents loaded with Au(III) were immersed into 0.1 mol L^−1^ HCl solution with a mass concentration of 4% thiourea. Then the regenerated adsorbent was washed 3 times with distilled water, and be served as the next adsorption/desorption cycle.

## Results and Discussion

### Characterization of Adsorbents

**Figure 1:**
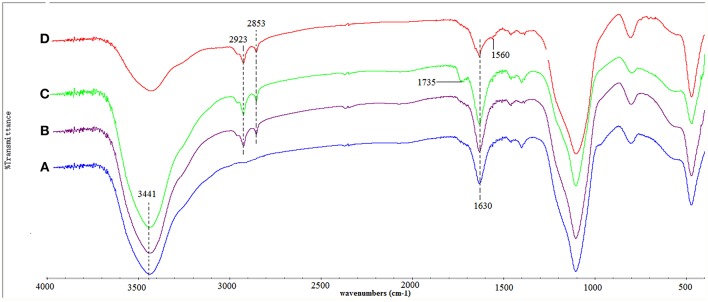
Infrared spectra of silica-gel **(A)**, SG-TETA **(B)**, SG-TETA-MA **(C)**, and SG-TETA2 **(D)**.

The FT-IR spectra of silica-gel, SG-TETA, SG-TETA-MA, and SG-TETA2 were showed on Figure 1. Silica-gel (Figure 1A) shows some characteristic bands at 3,441 cm^−1^ (stretching vibrations of Si-OH), 1,102 cm^−1^of stretching vibration of Si-O-Si (Tian et al., 2010; Niu et al., 2014). In the spectrum of SG-TETA (Figure 1B), the new peaks appeared at 2,853 and 2,923 cm^−1^ were assigned to the symmetric and asymmetric CH_2_ bands, which indicated the successful attach of carbon chain of CPTS-TETA on the surface of silica-gel. In ester-terminated grafting samples SG-TETA-MA (Figure 1C), the absorption at about 1,735 cm^−1^ indicated the existence of ester bonds (–COOCH_3_) (Mahmoudalilou et al., 2018). The absorption peaks 1,735 cm^−1^ of ester bonds in TETA-terminated SG-TETA2 disappeared (Figure 1D) but the peaks at about 1,560 cm^−1^ (N-H bending/C-N stretching, amide II) and 1,654 cm^−1^ (C = O, amide I) appeared, which suggested that the form of amides (Hayati et al., 2017). Hence, it was concluded that silica-gel supported TETA-terminated hyperbranched dendrimer-like polymer were successfully prepared by the step by step progress.

**Figure 2:**
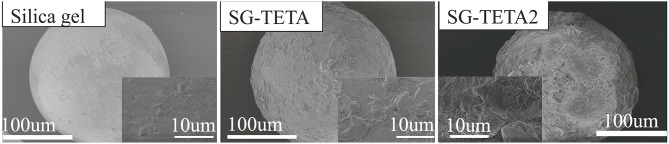
SEM images of silica-gel, SG-TETA, and SG-TETA2.

Figure 2 presents SEM images of Silica-gel, SG-TETA, and SG-TETA2, respectively. Obviously, the surface of silica-gel was relatively smooth. However, the surface of SG-TETA and SG-TETA2 became rougher after a series of reactions, which further suggested that the adsorbent was synthesized (Liu et al., 2013).

The contents of N in the SG-TETA, SG-TETA-MA, and SG-TETA2 demonstrated on Table 1, and the experimental content of amine groups be calculated. Compared to experimental content, the theoretical contents were many times greater than those. That might be due to intra-, inter- crosslinkings as well as steric hindrance, similar results were also found in our previous work (Qu et al., 2006; Zhang et al., 2015). After the Michael addition reaction between SG-TETA and MA, the functional groups content obviously decreased, and then increased after the amidation reaction. This is notably different from the results of series of silica-gel based DETA-terminated hyperbranched dendrimer-like polymer (Zhang et al., 2015). As one might expect, the long-chain of TETA with better stretch and flexibility had a positive effect on increasing the functional groups content of SG-TETA2. Moreover, compared to the porous structure parameters of SG-DETA2 in Table 1, SG-TETA2 not only retain existing pores and also increase new pores relying on nice stretch and flexibility of long-chain polyamine TETA.

**Table 1:** Elemental analysis results and porous structure parameters of Silica-gel, SG-TETA, SG-TETA-MA, SG-TETA2.

**Materials**	***N* (%)**	***N* (mmol g^**−1**^)**	**Amine groups content (mmol g**^**−1**^**)**		**BET surface area (m^**2**^ g^**−1**^)**	**BJH desorption cumulative volume of pores (cm^**3**^ g^**−1**^)^**a**^**	**BJH desorption average pore diameter (nm)**
			**Theoretical**	**Experimental**			
Silica-gel	-	-	-	-	429.92	0.97	7.80
SG-TETA	3.68	2.62	3.28	0.66	260.90	0.58	6.51
SG-TETA-MA	2.60	1.86	1.98	0.46	238.89	0.48	5.90
SG-TETA2	3.78	2.70	4.64	0.68	260.32	0.53	6.09

a *The BJH Desorption cumulative volume v of pores between 1.7 and 300 nm diameter*.

### Effect of pH on Adsorption of Au(III)

**Figure 3:**
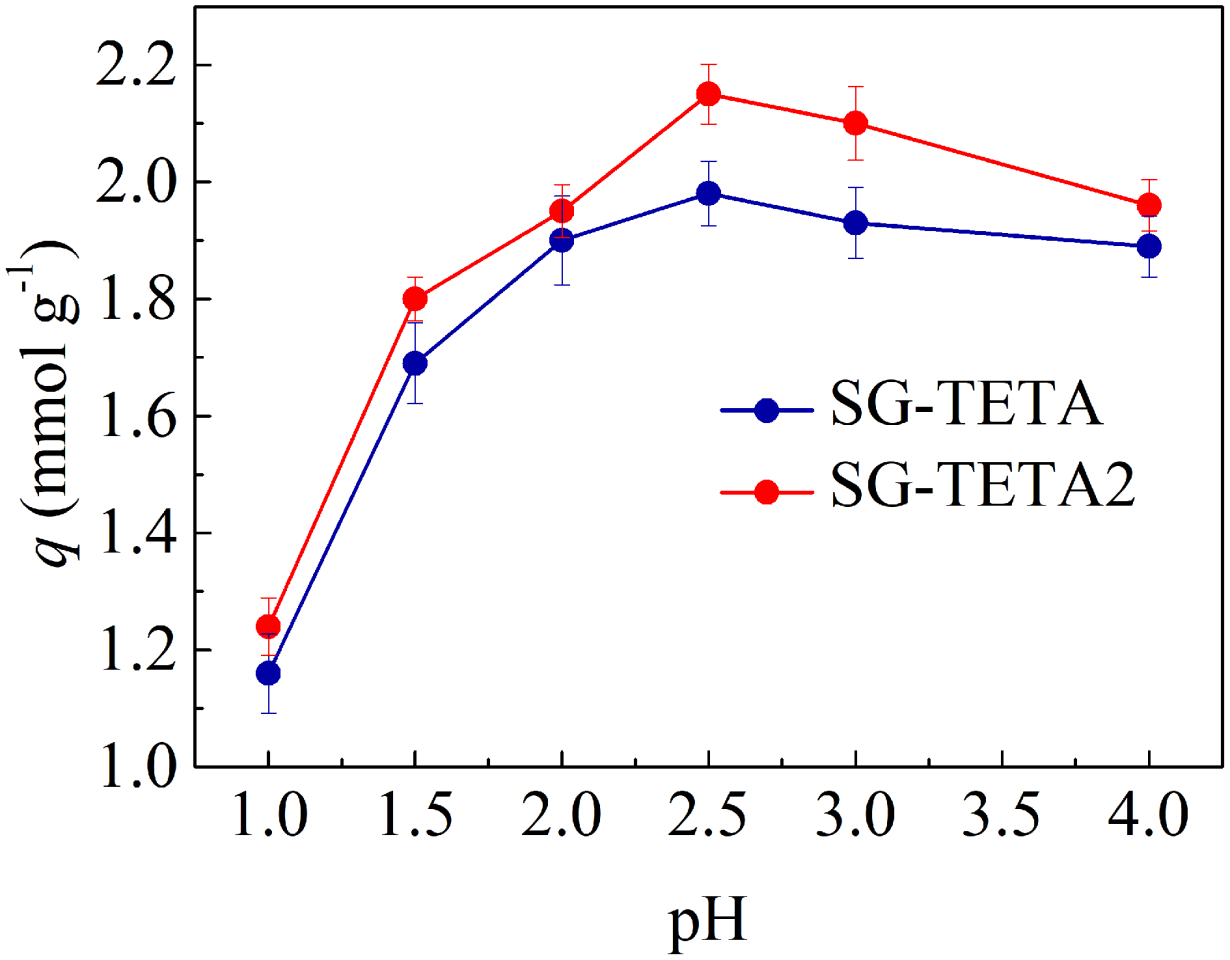
Effect of pH on the adsorption of Au(III) on SG-TETA and SG-TETA2 (initial concentration of Au(III) solution: 2.428 mmol L^−1^; 25°C).

According to the surface properties of adsorbents and gold attribute, the experimental range of pH was chosen from 1.0 to 4.0. The desired pH was adjusted with HCl and NaOH solutions. Figure 3 displayed the effect of pH on the adsorption of SG-TETA and SG-TETA2 for Au(III). With the increasing of pH, the adsorbed quantity increased first and attained the maximum at pH 2.5. The [AuCl_4_]^−^ complex ion are stabilized in highly acidic conditions, and nitrogen atoms of the functional group were positively charged through protonation at the same time (Ogata and Nakano, 2005). Hence, the electrostatic attraction would dominate under selected conditions. The lower adsorption at pH 1.0 was because of competition from Cl^−^ and [AuCl_4_]^−^. But the OH^−^ substituted some Cl^−^ in the [AuCl_4_]^−^ to form the [AuCl_4−n_(OH)_n_]^−^ complex ions (0 ≤ n ≤ 4.0) under weak acidic environment (Ando et al., 2018). Thus, with pH from 2.5 to 4.0 increasing, chloride-hydroxide complexes of Au(III) formed because of the reduction of protonated amines and hydroxide substitution reaction proceeds and the adsorption decreased gradually (Zhen et al., 2016). On the other hand, Au(III) ions are more likely to coordinate with lone pairs of electron of nitrogen on primary and secondary amines (Ahamed et al., 2013). The adsorption capacities of SG-TETA2 (2.15 mmol g^−1^) for Au(III) were slightly above SG-TETA's adsorption capacities (1.98 mmol g^−1^), which sufficient to explain a bit, the increase of generation was beneficial to adsorption. Compared to the DETA chain (the adsorption amounts of SG-DETA and SG-DETA2 were 1.95 and 1.98 mmol g^−1^) (Zhang et al., 2015), the long-chain TETA demonstrates an advantage on adsorption capacities.

### Adsorption Kinetics

**Figure 4:**
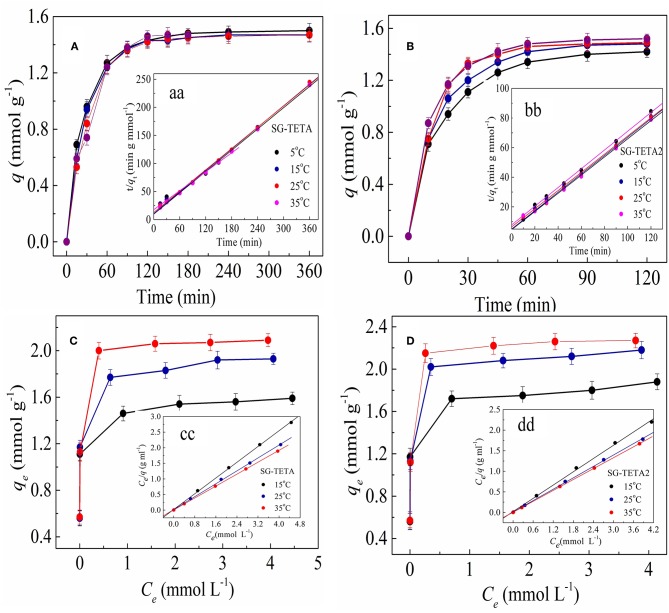
Adsorption kinetics for Au(III) on SG-TETA **(A)** and SG-TETA2 **(B)** (initial concentration of Au(III) solution: 2.428 mmol L^−1^; pH 2.5), pseudo-second-order kinetic plots for the adsorption of Au(III) onto on SG-TETA **(aa)** and SG-TETA2 **(bb)**; Isotherm for the adsorption of Au(III) on SG-TETA **(C)** and SG-TETA2 **(D)**, Langmuir isotherms for the adsorption of Au(III) on SG-TETA **(cc)** and SG-TETA2 **(dd)**.

Figures 4A,B shows the adsorption kinetic curves for Au(III) ions on silica-gel based dendrimer-like polymers adsorbents from 5 to 35°C. The adsorption process of SG-TETA for Au(III) proceeded rapidly first at all four temperatures, and reached the adsorption equilibrium at 180 min. And for SG-TETA2, the adsorption for Au(III) still proceeded rapidly at first but final equilibrium was reached after 90 min. By comparing them, the adsorption rate of SG-TETA2 for Au(III) is more rapidly, which may depend on the more amine groups and cavities in the second generation dendrimer-like functional groups with long-chain TETA. Rapid adsorption will beneficial to ensure economy efficiency of important practical significance. For the temperature effects, adsorption amounts of SG-TETA was affected slightly by temperature, while the adsorption amounts of SG-TETA2 had clear increase as the temperature went up under the experimental conditions. That is, the first generation SG-TETA seemed less susceptible to the temperature because the less crosslinking and little steric hindrance. However, more hydrogen bonds, the crosslinkings of amino, and blockages of the pores formed by introducing of more functional groups on the second generation adsorbents SG-TETA2 caused obstructions for Au(III) diffusion into the interior of holes at lower temperature. With the increasing of temperature, the stretch of long chains, the channels of molecular enlarges, and the combination for ions increases.

The kinetic data for Au(III) were analyzed by pseudo-first-order model (Konggidinata et al., 2017) and pseudo-second-order model (Nourmoradi et al., 2012) expressed as,

(2) $$\begin{array}{r} \text{ln }\frac{\left(q_{e}-q_{t}\right)}{q_{1}}=-k_{1}t \end{array}$$

(3) $$\begin{array}{r} \frac{t}{q_{t}}=\frac{1}{k_{2}q_{2}^{2}}+{\frac{t}{q}}_{2} \end{array}$$

Where, *q*_e_ (mmol g^−1^) and *q*_t_ (mmol g^−1^) are the amounts of Au(III) adsorbed at equilibrium and at time *t, k*_1_ (min^−1^) and *k*_2_ (g mmol^−1^ min^−1^) are the first order rate constant.

The kinetic parameters determined by the rate equations were summarized in Table 2. The fitting curves of pseudo-second-order (Figures 4aa,bb) were better than those of pseudo-first-order model, and the *R*^2^ values were more than 0.996 for Au(III) on SG-TETA and SG-TETA2, which means the Au(III) adsorbed rate determining step on SG-TETA and SG-TETA2 may be dominant by chemisorption but not mass transfer in solution (Nourmoradi et al., 2012; Monier and Abdel-Latif, 2013; Xiong et al., 2014; Qu et al., 2018). Also, the uptake capacities (*q*_e_) calculated from pseudo-second-order model were close to the experimental values, which demonstrated the availability of pseudo-second-order model for the adsorption. Furthermore, the initial adsorption rate (*h*, mmol g^−1^ h^−1^) and half-adsorption time t_1/2_ were obtained from *k*_2_ and *q*_e_ values using the Equations (4) and (5) (Wu et al., 2005), reflecting the speed of adsorption equilibrium were listed on Table 2.

(4) $$\begin{array}{r} h_{\;}=k_{2}q_{e}^{2} \end{array}$$

(5) $$\begin{array}{r} t_{1/2}=\frac{1}{k_{2}q_{e}} \end{array}$$

**Table 2:** Kinetic parameters for the adsorption of Au(III) onto SG-TETA and SG-TETA2 adsorbents at various temperatures.

**Adsorbents**	**T (°C)**	***q*_**e, exp**_ (mmol g^**−1**^)**	**Pseudo-first-order model**			**Pseudo-second-order model**				
			***q*_**e, cal**_ (mmol g^**−1**^)**	***k*_**1**_ (min^**−1**^)**	**R^**2**^**	***q*_**e, cal**_ (mmol g^**−1**^)**	***k*_**2**_ (g mmol^**−1**^ min^**−1**^)**	***H* (mmol g^**−1**^ min^**−1**^)**	***t*_**1/2**_ (min)**	**R^**2**^**
SG-TETA	5	1.47	2.30	0.042	0.9541	1.57	0.018	0.044	1252.15	0.9895
	15	1.47	1.27	0.026	0.9911	1.58	0.032	0.080	391.18	0.9968
	25	1.47	0.98	0.023	0.9651	1.56	0.039	0.095	270.16	0.9982
	35	1.50	0.86	0.020	0.9834	1.58	0.041	0.10	238.29	0.9992
SG-TETA2	5	1.42	1.20	0.049	0.9965	1.56	0.055	0.13	135.84	0.9986
	15	1.48	1.32	0.053	0.9956	1.62	0.059	0.15	109.46	0.9991
	25	1.49	0.97	0.053	0.9809	1.61	0.078	0.20	63.41	0.9976
	35	1.52	1.02	0.052	0.9980	1.63	0.082	0.22	55.97	0.9994

As can be seen from Table 2, the increased temperature can elevate the values of initial adsorption rate *h* due to the faster diffusion of Au(III) at high temperatures. Furthermore, *k*_2_ values increased and *t*_1/2_ decreased with the increase of temperature for both adsorbents, which suggested that higher temperature benefited to adsorption, that was, the adsorption needed shorter time to achieve equilibrium at higher temperature. The *k*_2_ values and *h* of SG-TETA2 had an obvious change when the temperature is >25°C, but little change observed was for SG-TETA. Also, less time was required for SG-TETA2 than for SG-TETA to achieve adsorption equilibrium under all treatment temperature. Thus, longer chain of functional group might make for enhancing the adsorption rate.

### Adsorption Isotherms

Adsorption isotherms can critically optimize the application to be established an adsorption process. The adsorption isotherms at experimental temperatures are presented in Figures 4C,D. As noted in Figures 4C,D, that the adsorption capacity increased with the initial Au(III) concentrations increase. The increase of adsorption capacity of SG-TETA and SG-TETA2 concerned with Au(III) concentrations can be considered a greater driving force formed by a higher concentration gradient pressure.

The isotherm adsorption data were modeled by Langmuir and Freundlich Equations (6) and (7), respectively, (Gurung et al., 2011).

(6) $$\begin{array}{r} \frac{C_{e}}{q_{e}}=\frac{1}{q_{\text{max}}K_{L}}+\frac{C_{e}}{q_{\text{max}}} \end{array}$$

(7) $$\begin{array}{r} \text{ln }q_{e}=\text{ln }k_{F}+\frac{1}{n}\text{ln }C_{e} \end{array}$$

where *q*_*e*_ and *C*_e_ are the equilibrium metal ion concentration on the adsorbent (mmol g^−1^) and in the solution (mmol L^−1^), *q*_max_ is the monolayer adsorption capacity of the adsorbent (mmol g^−1^), *K*_*L*_ and *k*_F_ are the Langmuir adsorption constant (L^3^ mg^−1^) and the Frendlich constant, and 1/*n* related to the adsorption driving force.

The correlation coefficient (*R*^2^≈0.99) for the Langmuir model (Langmuir curves see Figures 4cc,dd showed higher values than the Freundlich model tested (Table 3). Meanwhile, the data of the *q*_max_ of SG-TETA and SG-TETA2 for Au(III) calculated by this function were 2.10 and 2.27 mmol g^−1^ at 35°C, respectively, very close to the value experimentally obtained. While, low correlation coefficient values (*R*^2^ <0.95) of Freundlich isotherm deviated the application to the adsorption process. It was assumed that uniform adsorption energies with every Au(III) made equal affinity to the site and formed a monolayer adsorbate.

**Table 3:** Langmuir and Freundich isotherm adsorption constants for Au(III) on SG-TETA and SG-TETA2.

**Adsorbents**	**T (°C)**	**Langmuir**				**Freundlich**			**D-R**			
		***q*_**e**_ (mmol g^**−1**^)**	***q* (mmol g^**−1**^)**	***K*_**L**_ (mL mmol^**−1**^)**	$R_{\text{L}}^{2}$	***n***	***K*_**F**_ (mmol g^**−1**^)**	$R_{\text{F}}^{2}$	***q_***m***_* (mg g^**−1**^)**	***B* (mol^**2**^ J^**−2**^)**	***E* (kJ mol^**−1**^)**	**R^**2**^**
SG-TETA	15	1.59	1.58	32.00	0.9996	1.45	11.02	0.8559	1.64	4.55 × 10^−9^	10.48	0.8489
	25	1.93	1.95	32.35	0.9994	1.75	8.62	0.9093	2.02	5.59 × 10^−9^	9.46	0.9176
	35	2.11	2.10.	90.34	0.9999	1.93	7.51	0.9431	2.23	2.40 × 10^−9^	14.43	0.9566
SG-TETA2	15	1.88	1.86	25.98	0.9986	1.68	9.16	0.8900	1.93	5.45 × 10^−9^	9.57	0.8941
	25	2.18	2.17	43.52	0.9995	1.99	7.32	0.9481	2.33	6.59 × 10^−9^	8.71	0.9753
	35	2.27	2.27	81.50	0.9999	2.12	6.78	0.9485	2.50	6.87 × 10^−9^	8.53	0.9804

The isotherm data were also analyzed by Dubinin-Radushkevich (D-R) isotherm which was usually used to decide the physical or chemical adsorption. The linear form of the D–R isotherm Equation is 8 (Niu et al., 2014):

(8) $$\begin{array}{r} \text{ln }q_{e}=\text{ln }q_{\text{max}}-\beta \varepsilon^{2} \end{array}$$

where β is the activity coefficient (mol^2^ J^−2^) and ε is the Polanyi potential [ε = *RT* ln(1 + 1/*C*_e_)]. *E* (kJ mol^−1^) is mean free energy. The values of mean free energy (E, kJ mol^−1^) were calculated by using β values according to Equation (9):

(9) $$\begin{array}{r} E=\frac{1}{\sqrt{2\beta }} \end{array}$$

The adsorption is chemical adsorption in the range of 8 and 16 kJ mol^−1^ of *E* value while a physical adsorption the E value bellowing 8 kJ mol^−1^ (Dubinin, 1960). The results based on D-R were shown in Table 3, the *E* value of adsorption of SG TETA and SG TETA2 for Au(III) was >8.0 kJ mol, which indicated it was a chemical adsorption. But the *E* values are only slightly higher than 8 kJ mol^−1^, the author therefore believed that chemical chelating reactions play the main roles in the process of absorbing Au(III), and secondly by physical adsorption due to unique surface structure. Meanwhile, the bond energies of reversible chemical complexation <10 kJ mol^−1^ are beneficial for the separation (Yen et al., 2017). Thus, the desorption of Au(III) with SG-TETA and SG-TETA2 is supposed to readily proceed in our study.

Hence, the data simulated by Langmuir, Freundlich, and D-R demonstrated that the adsorption of SG-TETA and SG-TETA2 for Au(III) fitted well by Langmuir. That is, the adsorption was favorably monolayer adsorption and chemical adsorption played a major role at different temperatures.

The adsorption capacities of this study compared to other adsorbents are listed in Table 4. The adsorption capacity of SG-TETA and SG-TETA2 is clearly higher than most of the reported adsorbents. The ideal dendritic structure in SG-TETA and SG-TETA2 containing high density nitrogen and oxygen ligands could result in a desired adsorption capacity for Au(III). Compared with SG-DETA and SG-DETA2, the adsorption capacities of SG-TETA, and SG-TETA2 have a certain increase because the long-chain and less crosslinkings. Besides, the adsorption rate of SG-TETA and SG-TETA2 was faster than that of SG-DETA and SG-DETA2. For SG-TETA (Wang et al., 2013) prepared by heterogeneous, the adsorption was obvious lower than SG-TETA fabricated by homogeneous in this study. For much higher adsorption, the functional group mostly contain S atom, which can make adsorbed Au(III) more difficult to dilute (Pang and Yung, 2014; Gao et al., 2017). SG-TETA and SG-TETA2 had the higher adsorption amount and rate, and could be employed as a significant potential candidate for Au(III) uptake.

**Table 4:** Comparison of maximum adsorption capacities (*q*_max_) of some adsorbents for Au(III) from aqueous solution.

**Adsorbents**	**pH**	**Adsorption time**	**Au(III) adsorption capacity (mmol g^**−1**^)**	**References**
Silica-gel				This study
SG-TETA	2.5	4 h	2.10	
SG-TETA2	2.5	2 h	2.27	
Silica-gel				Zhang et al., 2015
SG-DETA	2.0	4 h	2.09	
SG-DETA2	2.0	4 h	2.12	
Silica-gel supported amino-terminated dendrimer-like polyamidoamine polymer				Qu et al., 2014
G1.0	3.0	24 h	1.48	
G2.0	3.0	24 h	2.45	
Silica gels functionalized with triethylenetetramine (SG-TETA)	3.29	1.5 h	1.43	Wang et al., 2013
Thioctic acid functionalized silica coated magnetite nanoparticles	5.0	4 h	1.45	Fadzilah et al., 2018
D301 resin functionalized with ethylenediamine	2.0	5 h	1.51	An et al., 2017
D301 resin functionalized with ethylenediaminethiourea	2.0	5 h	1.65	
Thiosemicarbazide functionalized corn bract	6.0	24 h	7.46	Lin et al., 2018
Thiourea modified alginate	1.0	48 h	8.47	Gao et al., 2017
Multiwalled carbon nanotubes	–	24 h	0.47	Pang and Yung, 2014
Oxidized multi-walled carbon nanotubes	2.0	1 h	0.32	Shaheen et al., 2015

### Thermodynamic Parameters

**Figure 5:**
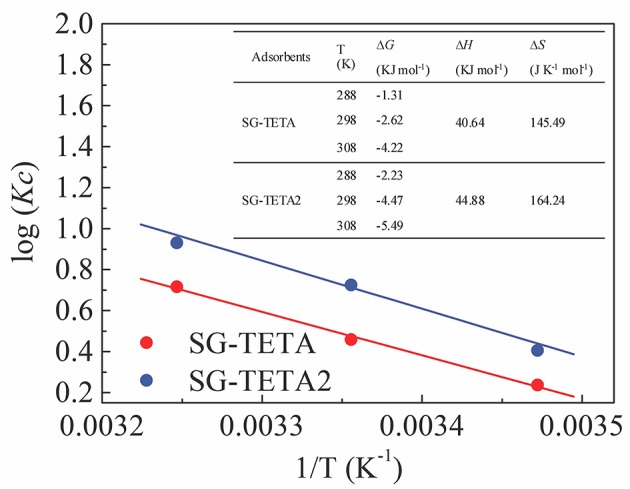
log*K*_*c*_ vs. 1/T plots and thermodynamic parameters for Au(III) adsorption on SG-TETA and SG-TETA2.

Figure 5 illustrated the thermodynamic parameters including Gibbs free energy changes (ΔG°), enthalpy change (ΔH°), and entropy change (ΔS°), which were based the following equations,

(10) $$\begin{array}{r} K_{c}=\frac{C_{Ae}}{C_{e}} \end{array}$$

(11) $$\begin{array}{r} \text{log }K_{c}=\frac{\Delta S}{2.303R}-\frac{\Delta H}{2.303RT} \end{array}$$

(12) $$\begin{array}{r} \Delta G^{\circ }=-RT\text{ ln }K_{c} \end{array}$$

where *C*_*Ae*_ and *C*_*e*_ (mg L^−1^) are the equilibrium concentrations of Au(III) ion on adsorbent and in solution, respectively, *K*_c_ is the thermodynamic equilibrium constant, *R* (KJ mol^−1^) is gas constant, *T* (K) is the absolute temperature, and Δ*G*° is the standard free energy change.

From Figure 5, the negative values of Δ*G*° indicated the adsorption was spontaneous and thermodynamically favored. The positive enthalpy (Δ*H*°) showed the adsorption was an endothermic process and as a result the adsorption capacity increased with an increase in temperature. The positive entropy (Δ*S*°) reflected an increase in the randomness at the interface of solid-liquid interface during the adsorption.

### Adsorption Mechanism

**Scheme 2:**
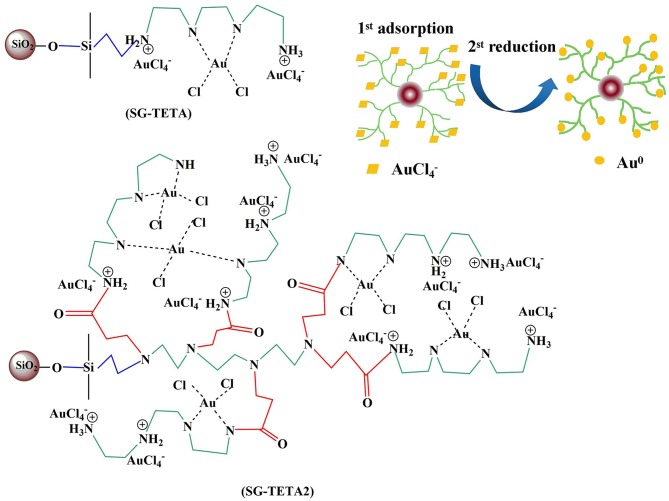
A suggested bonding mode of SG-TETA and SG-TETA2 of silica gel surface with favorable binding of Au(III) to amino group.

High utilization of functional groups on silica-gel-based dendrimer-like polymers containing long-chain triethylenetetramine could ensure a quick and efficient adsorption capability. Table 1 presented that the N contents of SG-TETA and SG-TETA2 were determined to be 2.62 and 2.70 (mmol g^−1^). Thus, N of adsorbents might form complex with Au (III) by 1.24:1 (SG-TETA) and 1.19:1 (SG-TETA2). Note that the utilization of N of the second-generation SG-TETA2 was better than that of SG-TETA because of long and flexible triethylenetetramine chains. Furthermore, the crosslinkings was believed to be not obvious that the [AuCl_4_]^−^ could enter into inside of the functional groups and adsorb on the surface. This result also indicated that steric hindrance is one of the main causes of low functional groups contents. It was the steric hindrance of TETA2 that give the optimal channel and capture metal ions. Three possible mechanisms: electrostatic interaction, chelation and physical adsorption, were proposed to govern the adsorption for Au(III) by the adsorbents containing nitrogen and oxygen ligands from HCl media (Adhikari et al., 2013). According to the above facts, we proposed an Au(III) ion may chelate with two nitrogen atoms or electrostatic adsorption with one nitrogen atom of PAMAM dendrimer including not only external but also internal ones. A proposed complexion of amino group on SG-TETA and SG-TETA2 for Au(III) was presented in Scheme 2.

**Figure 6:**
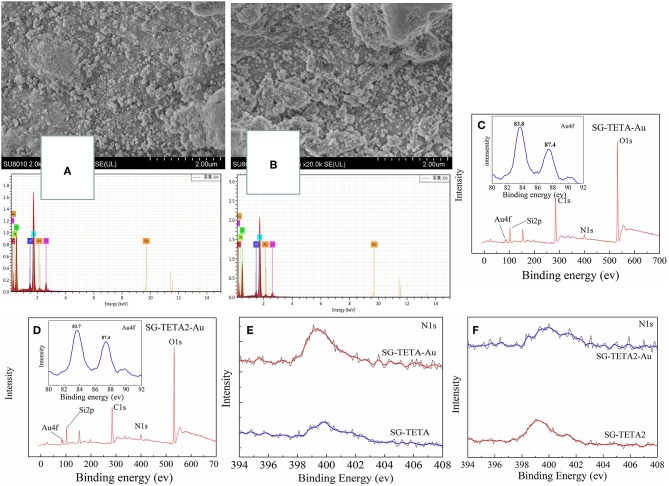
SEM and EDX images and of SG-TETA **(A)** and SG-TETA2 **(B)** before and after adsorption of Au(III); XPS spectra of SG-TETA **(C)** and SG-TETA2 **(D)** before and after adsorption; Au 4f spectra of SG-TETA **(E)** and SG-TETA2 **(F)** before adsorption (red) and after adsorption (blue).

To gain further insight into the mechanism, Figures 6A,B showed the SEM and EDX images of SG-TETA and SG-TETA2 after adsorption. Obviously, there were plenty of grains distributed on the surface of the adsorbents, which might be because Au(III) ion were reduced to Au(0) after adsorption. The EDX lines at 2.2 keV indicated existence of the gold nanoparticles. Peaks were also noted around 8.2 keV which indicates the presence of gold atoms (Shah et al., 2017). X-ray photoelectron spectroscopy was further used to study the surface chemical compositions of the as-prepared and Au(III) ions adsorbed silica gel based dendrimer-like polymer. Figures 6C,D shows the two survey spectra manifesting surface chemical compositions of the adsorbents before and after adsorption of Au(III) ions. After adsorption, the appearance of Au on the surface of SG-TETA and SG-TETA2 was obviously observed. The peaks at 87.4 eV (Au 4f 5/2) and 83.7 eV (Au 4f 7/2) are related to Au(0), which indicated that Au(III) was reduced to Au(0) by the SG-TETA and SG-TETA2. Also the slight peaks around at 90.0 eV (Au 4f 5/2) and 82.0 eV (Au 4f 7/2) were assigned to Au(III), which demonstrated the formation of [NH_2_-Au(III)] (Li et al., 2013). Therefore, the adsorbed Au(III) ions might exist in two forms: oxidation state zero and positive three, indicating that NH_2_ in the functional groups could not only coordinate with Au(III) can also reduce Au(III) (Wang et al., 2010). Bonding energy peaks at 399.2 eV in Figures 6E,F related to free amino (-NH_2_), but the peak shift to higher binding energy after adsorption of Au(III), which illustrated the -NH_2_ group can attract [AuCl_4_]^−^ effectively (Gao et al., 2017). All the results above showed that the adsorption for gold on the surface of SG-TETA and SG-TETA2 was considered the adsorptive-reduct process.

### Adsorption Selectivity

A series of binary ions coexistent systems, Au(III)-Cu(II), Au(III)-Pb(II), and Au(III)-Ni(II), were investigated to evaluate the adsorption selectivity of SG-TETA and SG-TETA2 for Au(III) and the results were listed in Table 5. From Table 5, Au(III) selected adsorption preferentially by SG-TETA and SG-TETA2 from the binary systems, and selective coefficients both are infinite, indicating that SG-TETA, and SG-TETA2 for adsorption Au(III) showed excellent selectivity.

**Table 5:** The adsorption selectivity of adsorbents for Au(III) (Au(III) concentration: 2.428 mmol L^−1^; concentration of coexisting metal ions: 2.5 mol L^−1^; pH = 2.5; T = 25°C.

**Adsorbents**	**System**	**Metal ions**	**Adsorbents capacity (mmol g^**−1**^)**	**Selective coefficient ^**a**^**
SG-TETA	Au(III)-Cu(II)	Au(III)	1.16	α_Au(III)/Cu(II)_ = ∞
		Cu(II)	0	
	Au(III)-Ni(II)	Au(III)	1.29	α_Au(III)/Ni(II)_ = ∞
		Ni(II)	0	
	Au(III)-Pb(II)	Au(III)	1.20	α_Au(III)/Pb(II)_ = ∞
		Pb(II)	0	
SG-TETA2	Au(III)-Cu(II)	Au(III)	1.41	α_Au(III)/Cu(II)_ = ∞
		Cu(II)	0	
	Au(III)-Ni(II)	Au(III)	1.52	α_Au(III)/Ni(II)_ = ∞
		Ni(II)	0	
	Au(III)-Pb(II)	Au(III)	1.48	α_Au(III)/Pb(II)_ = ∞
		Pb(II)	0	

### Desorption and Regeneration Performance

The strong complexing agent, thiourea [(NH_2_)_2_CS)] with lower toxicity offered S and N ligands, which can leach gold by forming a complex in acidic media based the reaction (Dwivedi et al., 2014):

(13) $$\begin{array}{r} \text{A}{\text{u}}^{0}+2\text{SC}{\left(\text{N}{\text{H}}_{2}\right)}_{2}\to \text{Au}{\left[\text{CS}{\left(\text{N}{\text{H}}_{2}\right)}_{2}\right]}_{2}^{+}+{\text{e}}^{-} \end{array}$$

**Figure 7:**
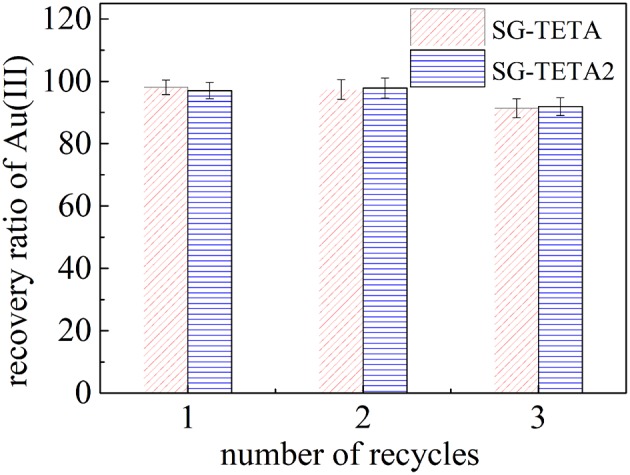
Repetitive adsorption and desorption of Au(III) SG-TETA and SG-TETA2.

In this study, the adsorption-desorption recycles of SG-TETA and SG-TETA2 were repeated for 3 times by using 4% thiourea/0.1 mol L^−1^ HCl solution (Figure 7). The recovery of Au(III) remained above 91% in 3 cycles indicated the good adsorption of reused SG-TETA and SG-TETA2 which can remain relatively stable. The repeated adsorption-desorption results demonstrate that the adsorption sites on the surface of the SG-TETA and SG-TETA2 are reversible. Thus, these triethylenetetramine terminal hyperbranched dendrimer-like polymer adsorbents possess the most potential as a gold recovery material due to the good adsorption amount, high selectivity, and stable regeneration.

## Conclusions

The adsorption properties of silica-gel-based dendrimer-like polymers functionalized by long-chain triethylenetetramine in water were investigated. The utilization of functional long-chain TETA was remarkable and therefore could remove Au(III) much effectively. The adsorption of the SG-TETA2 rapidly reached equilibrium within 90 min, while for the SG-TETA equilibrium was reached after 120 min. The fast establishment of the equilibrium for Au(III) in a short time showed the effectiveness of the adsorbent for recovery. The adsorption mechanism of SG-TETA and SG-TETA2 for Au(III) is composed by adsorption and reduction. SG-TETA and SG-TETA2 also showed excellent selectivity for the adsorption for Au(III) in a co-existed metal ion system. The long-chain TETA was beneficial for adsorption due to more sites and its flexibility.

## Data Availability

The datasets generated for this study are available on request to the corresponding author.

## Author Contributions

YiZ carried out experiments and wrote the manuscript. RQ designed experiments and analyzed results. TX and YuZ carried out adorption experiments. CS, CJ, and WY characterized and analyzed experimental results.

### Conflict of Interest Statement

The authors declare that the research was conducted in the absence of any commercial or financial relationships that could be construed as a potential conflict of interest.
